# Quantum Graphene Asymmetric Devices for Harvesting Electromagnetic Energy

**DOI:** 10.3390/nano14131114

**Published:** 2024-06-28

**Authors:** Mircea Dragoman, Adrian Dinescu, Martino Aldrigo, Daniela Dragoman

**Affiliations:** 1 National Institute for Research and Development in Microtechnologies (IMT), Strada Erou Iancu Nicolae 126A, 077190 Voluntari, Romania; mircea.dragoman@imt.ro (M.D.); adrian.dinescu@imt.ro (A.D.); 2 Physics Faculty, University of Bucharest, P.O. Box MG-11, 077125 Bucharest, Romania; daniela.dragoman@unibuc.ro; 3 Academy of Romanian Scientists, Strada Ilfov, Nr. 3, 050044 Bucharest, Romania

**Keywords:** diode, electromagnetic radiation, energy harvesting, graphene, quantum technologies

## Abstract

We present here the fabrication at the wafer level and the electrical performance of two types of graphene diodes: ballistic trapezoidal-shaped graphene diodes and lateral tunneling graphene diodes. In the case of the ballistic trapezoidal-shaped graphene diode, we observe a large DC current of 200 µA at a DC bias voltage of ±2 V and a large voltage responsivity of 2000 *v*/*w*, while in the case of the lateral tunneling graphene diodes, we obtain a DC current of 1.5 mA at a DC bias voltage of ±2 V, with a voltage responsivity of 3000 *v/w*. An extended analysis of the defects produced during the fabrication process and their influences on the graphene diode performance is also presented.

## 1. Introduction

**Figure 1:**
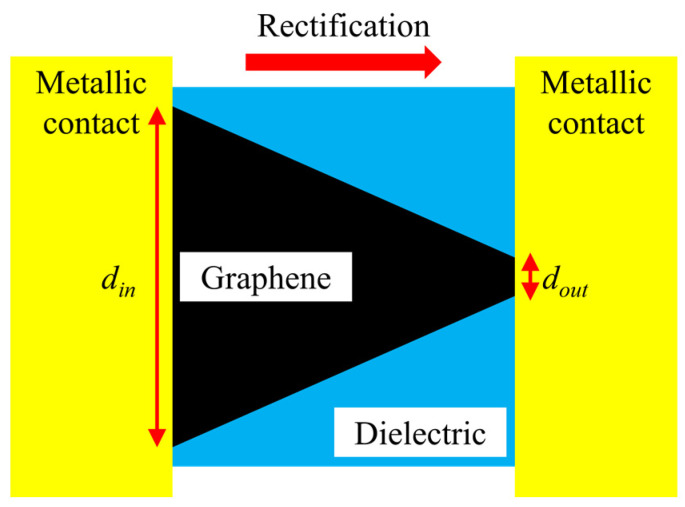
Schematic representation of the geometric graphene diode with a trapezoidal shape.

Geometric diodes based on graphene monolayers have been studied both theoretically [1] and experimentally [2,3,4,5,6,7] to produce the rectification of an incoming high-frequency signal, taking into account that any doping could irreversibly damage the single atomic layer of carbon. The first type of geometric graphene diode had a trapezoidal shape, with a “neck” (denoted as *d_out_*) of a few tens of nanometers and a “shoulder” of around 100 nm (denoted as *d_in_*, see Figure 1) working in a ballistic transport regime.

A rectification is produced due to the tapered geometry of the graphene monolayer. The ballistic transport in graphene is strongly dependent on the substrate on which the graphene is transferred. The mean free path at room temperature is around 400–500 nm when the substrate is silicon dioxide (SiO_2_) [8,9] and increases beyond 1 µm when the graphene is transferred on hexagonal boron nitride (h-BN) [10,11]. Moreover, in the case of the ballistic regime, the cutoff frequency is limited solely by the traversal time of carriers which, in gated graphene at a normal incidence, is the ratio of the length of the device and the Fermi velocity *v_F_*, with *v_F_* reaching about 3 × 10^6^ m/s in graphene [12]. In our case, this translates into a THz cutoff frequency.

There is an increased interest in the simulation of geometric graphene diodes, mentioning here only the latest references [13,14,15]. These efforts at both the theoretical and experimental level are due to the promising applications of this type of devices at THz frequencies and for energy harvesting in the infrared (IR) region. THz detection using various geometric graphene diodes or field-effect transistors (FETs) was experimentally demonstrated in [16,17,18,19], whereas IR harvesting solutions were reported in [20,21,22]. Moreover, these devices have the advantage of requiring merely a specific geometric shape (as their name itself suggests), which can be engineered to attain the desired performance in terms of current level, nonlinearity, cutoff frequency, etc.

Very recently, a new area of graphene-based electronic applications has been opened, in which it has been demonstrated experimentally that ballistic FETs, having a graphene monolayer channel with a trapezoidal shape as in Figure 1, exhibit a subthreshold swing (SS) well below 60 mV/decade, meaning that ballistic geometric graphene diodes and transistors can be used in low-power applications [23,24,25,26,27,28,29,30]. The idea of the geometric asymmetry of the graphene layer could be profitably exploited also for geometric contacts in zero-bias radiofrequency (RF)/millimeter-wave and light detectors, as conducted in [31,32,33,34,35,36,37,38].

**Figure 2:**
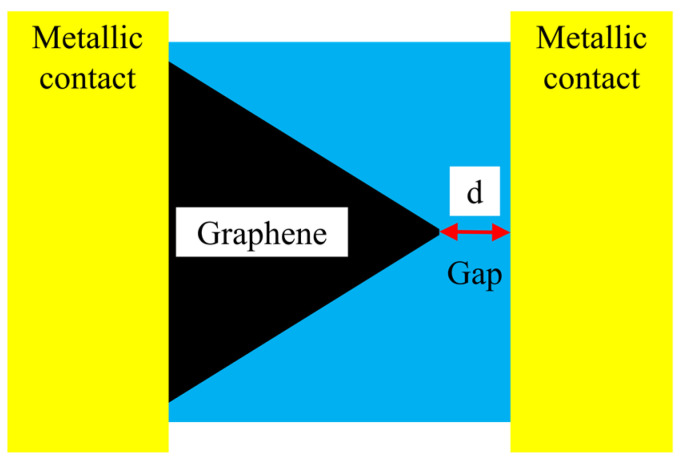
Schematic view of the geometrically enhanced graphene tunnel diode with a lateral air gap.

Recently, it was discovered that the tunneling current is enhanced in geometric graphene diodes through a nanosized gap that is placed laterally near a metallic contact, as it is shown in Figure 2 [39]. It is a lateral tunneling metal-insulator-metal (MIM) diode, where the insulator (or dielectric) is air. In this way, this lateral tunneling graphene diode can be elevated as a nanoscale air channel device [40]. The electric field is significantly enhanced at the sharp tip of graphene [41], and thus, the tunneling efficiency is increased by reducing the barrier height due to the confinement of the electric field. Since the gap has a width of 50 nm in [39], and of 20 and 30 nm in [41], the tunneling time is very short, i.e., around 10–20 fs, which translates into a cutoff frequency of tens of THz. These results make lateral tunneling MIM diodes the fastest to date and open the possibility of harvesting the mid-infrared (mid-IR) spectrum or, in other words, to convert mid-IR radiation into a DC signal, thus realizing solar cells working day and night [42]. The latter possibility stems from the big portion of IR power that is emitted by the sun but is unavoidably lost by conventional solar cells.

The fabrication of this type of diode is much simpler than other tunneling graphene diodes that are vertical resonant tunneling diodes (RTDs), such as those reported in [43,44,45], which are formed by stacked h-BN and graphene monolayers transferred onto a doped Si/SiO_2_ serving as a gate. In fact, they are MIM-like structures, where the role of the metal is played by graphene and the insulator is h-BN. Stacked MoS_2_ monolayers [46] and black phosphorus (BP)/aluminum oxide (Al_2_O_3_)/BP were also used to fabricate RTDs [46,47,48].

This intensive research of two-dimensional (2D) material-based tunneling diodes is supported by the fact that RTD is a single-electron device able to generate and detect frequencies in the THz range (for a recent review, please see [49]). However, none of the devices mentioned above were fabricated at the wafer scale (as conducted in the case of semiconductor-based tunneling diodes) but instead using flakes of various 2D materials.

Thus, the role of this paper is to narrow the large gap between the tunneling diodes based on 2D materials and semiconductors by fabrication and testing at the wafer scale of trapezoidal-shaped and tunneling (i.e., with a lateral gap) graphene diodes, and to study their physical properties.

## 2. Fabrication of Quantum Graphene Diodes at the Wafer Scale

**Figure 3:**
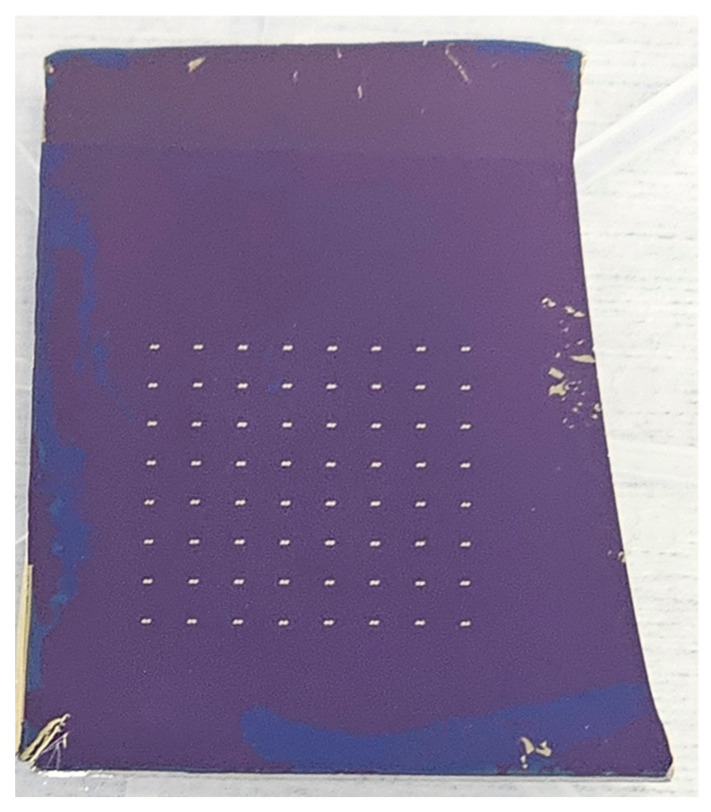
Optical image of the quantum graphene chip.

The quantum graphene diodes were fabricated on a doped Si/SiO_2_ 4-inch wafer. The thickness of the Si wafer is 525 µm, while the thermally grown SiO_2_ has a thickness of 300 nm. The graphene monolayer was grown by means of a chemical vapor deposition (CVD) process and then transferred on the Si/SiO_2_ substrate by Graphenea (San Sebastian, Spain). Raman spectroscopy has shown that almost 80% of the wafer’s surface was covered with a graphene monolayer, the rest being areas with graphene multilayers containing 4–6 monolayers and grain boundary defects. The fabrication consists in the following steps: (i) patterning the graphene channel by electron-beam lithography (EBL) and reactive ion etching (RIE); (ii) patterning the shapes of the quantum graphene diodes using EBL; and (iii) the patterning, metallization, and liftoff of the Cr (5 nm)/Au (240 nm) metallic contacts deposited using an e-beam process. We fabricated two identical graphene chips cut from the wafer, and the optical image of a chip is presented in Figure 3. In total, we fabricated 128 quantum diodes, 64 for each chip in two fabrication runs.

**Figure 4:**
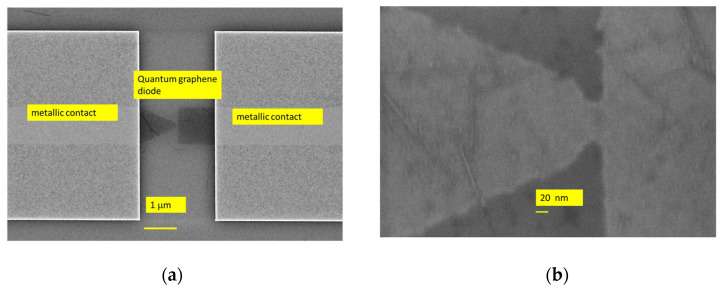
(**a**) SEM picture of a quantum graphene diode with metallic contacts; (**b**) SEM picture of a trapezoidal graphene diode.

**Figure 5:**
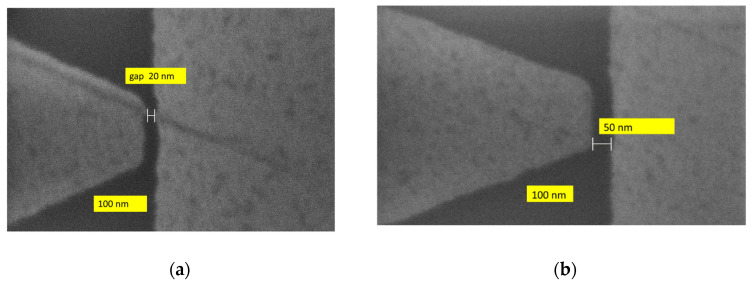
SEM pictures of graphene tunneling diodes with an air gap of (**a**) 20 nm and (**b**) 50 nm.

The first four lines are occupied with trapezoidal-shaped diodes (see Figure 1) and the last four ones with graphene tunnel diodes (see Figure 2) with different dimensions of the air gap, each column containing a specific width, i.e., 10 nm, 20 nm, 30 nm (two columns), 40 nm, 50 nm, and 60 nm, plus one column with trapezoidal-shaped diodes (i.e., with no air gap). The SEM image of a quantum graphene diode with metallic contacts is displayed in Figure 4a, while the SEM images of a single quantum diode are shown in Figure 4b (trapezoidal shape) and Figure 5a,b (graphene tunneling diodes).

## 3. Measurements and Discussion

The current-voltage (I-V) measurements were performed using a Keithley SCS 4200 station (Beaverton, OR, United States) at room temperature. Since the thickness of the substrate is 525 µm for Si and 300 nm for SiO_2_, the back gate voltage works at 30–40 V by slightly changing the current by 10–20%. Therefore, to study the potential harvesting properties of a two-terminal device, the back gate was intentionally disaffected from the measurements, since we did not consider here a back-gate transistor, but we investigated the DC properties of harvesting devices working at nearly 0 V, i.e., in unbiased conditions. The entire probe station for on-wafer measurements was placed inside a Faraday cage and was connected to the station via low-noise amplifiers. All the devices were measured, but only 10% of them provided a similar response.

**Figure 6:**
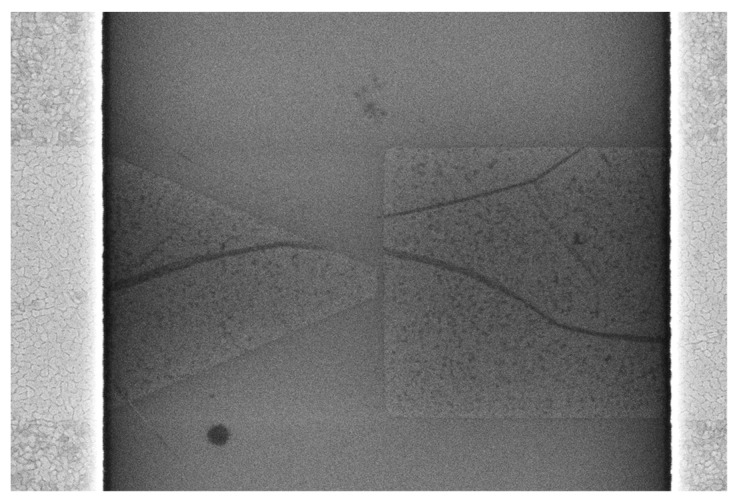
SEM picture showing cracks in a graphene diode.

Therefore, we performed an SEM analysis in parallel with the electrical measurements to identify the issues producing the loss of electrical functionalities of quantum graphene devices. These errors originate from several sources. The first are the defects in graphene, which are accentuated when the graphene is etched by RIE, since this technique often produces large cracks in graphene that interrupt the current flow (see Figure 6). More than 50% of the non-functioning devices show such cracks. A second major error occurs from graphene debris over the graphene diodes produced by the e-beam process and that can be observed in 20% of the total number of devices. Edge roughness effects in graphene could be found well explained in [50] based on TEM measurements and DC measurements at 4K. However, even if the etching is performed with RIE in place of plasma oxygen, the effects of e-beam lithography itself (i.e., misalignments and proximity) cannot be avoided due to the very small dimensions. In [50], the edge effects play a certain role in the transport measurements performed at very low temperatures and high magnetic fields, but in our case, the coherence inherent to ballistic transport is preserved even if a few scattering events happen (i.e., in a quasi-ballistic regime) since the key parameter for coherence survival is the phase coherence length [51]. Therefore, here, the main source of the defects is the graphene etching based on plasma oxygen, which produces cracks in the monolayer, and the debris resulting from this operation seriously affects the yield and the functionalities of the devices. The rest of the defects arise from the exfoliation of the metallic deposition during measurements. All these defects destroy either the ballistic carrier transport or tunneling mechanism in graphene diodes.

The performance of the graphene diodes is investigated, starting from the measured I-V characteristics at room temperature, and then we extract the differential resistance R_D_ (Ω), the nonlinearity χ (a.u.), the voltage responsivity β (*v*/*w*), and the sensitivity γ (V^−1^), which are defined in the literature, for example, in [52]. In detail, we use the following expressions, which are valid, irrespective of the nonlinear device under test and of the frequency:

(1) $$R_{D}=1/\left(\partial I/\partial V\right)$$

(2) $$\chi =\left(\partial I/\partial V\right)/\left(I/V\right)$$

(3) $$\beta =0.5R_{D}\gamma$$

(4) $$\gamma =\left(\partial^{2}I/\partial V^{2}\right)/\left(\partial I/\partial V\right)$$

**Figure 7:**
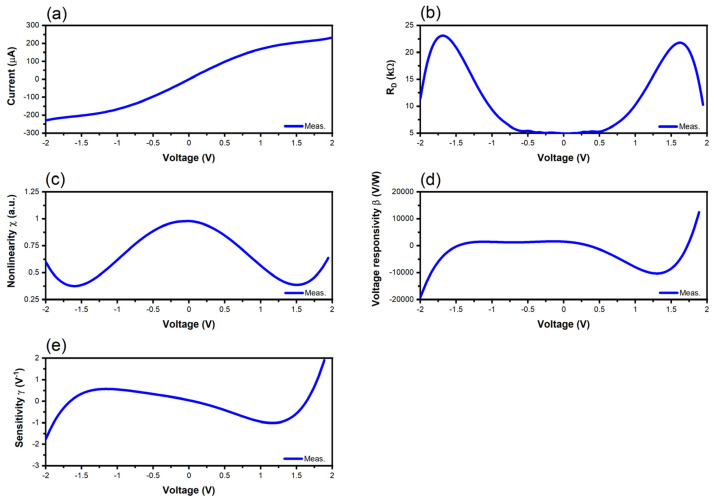
Measured and extracted performance of the trapezoidal graphene diode: (**a**) I-V curve; (**b**) differential resistance; (**c**) nonlinearity; (**d**) voltage responsivity; (**e**) sensitivity.

The I-V dependence at room temperature of the trapezoidal graphene diode, its differential resistance R_D_, its nonlinearity χ, its voltage responsivity β, and its sensitivity are represented in Figure 7. We can observe a large DC current of 200 µA at a DC bias voltage of ±2 V, a large responsivity of 2000 *v*/*w*, and a differential resistance varying in the range 5–25 kΩ.

**Figure 8:**
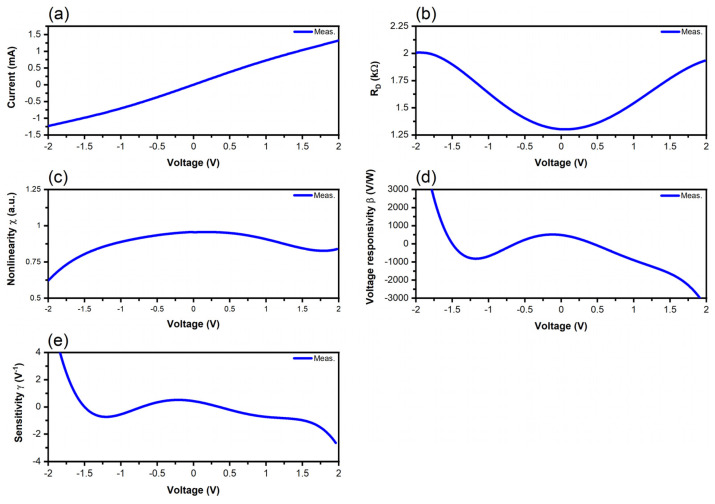
Measured and extracted performance of the tunneling graphene diode: (**a**) I-V curve; (**b**) differential resistance; (**c**) nonlinearity; (**d**) voltage responsivity; (**e**) sensitivity.

The I-V dependence at room temperature of the tunneling graphene diode, its differential resistance R_D_, its nonlinearity χ, its voltage responsivity β, and its sensitivity are represented in Figure 8. This diode exhibits the highest values of the DC current and the lowest values of R_D_ (i.e., not exceeding 2 kΩ), thus making it the most suitable for the integration with a matching network to guarantee the maximum power transfer of the harvested energy. The nonlinearity is less than 1, but the voltage responsivity attains values between −3000 and 3000 *v*/*w*, while the sensitivity is the highest, i.e., between −3 and 4 V^−1^. The fact that tunneling diodes exhibit better performance than trapezoidal diodes is explained by the fact that in the trapezoidal diodes, the carriers reflect at the boundaries of the trapezoidal shape; hence, quantum interferences are produced and they decrease the current [53]. In contrast, in the case of the tunneling graphene diode, direct tunneling and Fowler-Nordheim tunneling occur simultaneously, thus producing together a high DC current measured at rather low voltages [39].

We stress here that to obtain such a performance, special measurement conditions were adopted. The wafers with the fabricated diodes were kept in vacuum and then used in ambient conditions only for measurements; eventually, they were further preserved in a vacuum. The reason is that the ambient conditions or any technological imperfection could easily destroy the ballistic transport or tunneling phenomena.

**Figure 9:**
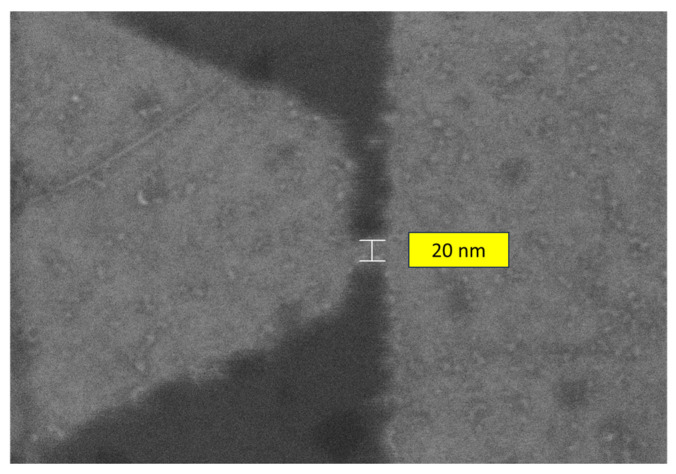
SEM picture of a graphene tunneling diode not completely etched.

To illustrate this last situation, we have measured several tunneling diodes where the gap is not completely etched and graphene has defects, typically poly(methyl methacrylate) (PMMA) residues (Figure 9).

**Figure 10:**
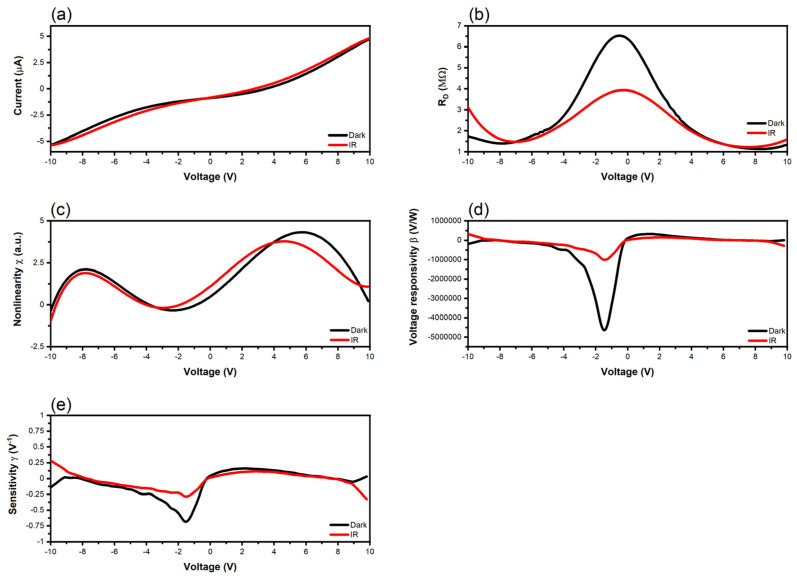
Measured and extracted performance of the tunneling graphene diode with defects: (**a**) I-V curve; (**b**) differential resistance; (**c**) nonlinearity; (**d**) voltage responsivity; (**e**) sensitivity.

Finally, the results of the I-V dependence at room temperature of the tunneling graphene diode with defects, its differential resistance R_D_, its nonlinearity χ, its voltage responsivity β, and its sensitivity are represented in Figure 10 in dark and illuminated conditions, the latter using an IR source AvaSpec NIR 256-2.2 (Apeldoorn, The Netherlands), which covers the wavelength spectrum 1.6–2.1 µm, and with a power of 27 VA. In order to verify if a breakdown occurs when pushing the DC bias to higher values, we consider the voltage interval [−10, 10] V; nevertheless, the focus is always between −2 V and 2 V, where the diode is supposed to work under a low-incident power level. The current is about three orders of magnitude lower compared with the graphene tunneling diode without defects, and the differential resistance increases with three orders of magnitude. Although the responsivity and sensitivity are high, i.e., −4.6 × 10^6^ *v*/*w* and −0.7 V^−1^ in dark conditions, respectively, and about −1 × 10^6^ *v*/*w* and −0.29 V^−1^ under IR illumination, the performance is seriously degraded compared with a graphene tunneling diode without defects. We notice here that in geometric diodes, a negative voltage responsivity is associated to an n-type behavior, whereas a p-type behavior generates a positive voltage responsivity [54].

## 4. Conclusions

In this work, we presented the characterization of ballistic and tunneling graphene diodes fabricated at the wafer level. The fact that tunneling diodes exhibit better performance than trapezoidal diodes is explained by the fact that in the trapezoidal diodes, the carriers reflect at the boundaries of the trapezoidal shape; hence, quantum interferences are produced and they decrease the current. In contrast, in the case of the tunneling graphene diode, direct tunneling and Fowler-Nordheim tunneling occur simultaneously, thus producing together a high DC current measured at rather low voltages. We have also studied the degradation of the performance of the graphene tunneling diodes when defects appear within their air gap. Thus, the lateral tunneling graphene diode is a strong candidate for harvesting electromagnetic energy from microwave to infrared radiation. The main advantage of our investigation is the fact that we found the fastest electronic devices to date for harvesting electromagnetic waves. On the other hand, the main limitations come from fabrication since there are a couple of sources of defects that drastically reduce the final yield. In perspective, we aim at integrating these devices with THz antennas to create whole rectenna systems working up to infrared.

## Data Availability

The original contributions presented in the study are included in the article, and further inquiries can be directed to the corresponding author.
